# Re-calibration of coronary risk prediction: an example of the Seven Countries Study

**DOI:** 10.1038/s41598-017-17784-2

**Published:** 2017-12-14

**Authors:** Paolo Emilio Puddu, Paolo Piras, Daan Kromhout, Hanna Tolonen, Anthony Kafatos, Alessandro Menotti

**Affiliations:** 1 Department of Cardiovascular, Respiratory, Nephrological, Anesthesiological and Geriatric Sciences, Sapienza University of Rome, Rome, Italy; 2 Division of Human Nutrition, Wageningen University, Wageningen, The Netherlands and Department of Epidemiology, University Medical Center Groningen, University of Groningen, Groningen, The Netherlands; 3 Department of Public Health Solutions, National Institute for Health and Welfare, Helsinki, Finland; 4 Department of Social Medicine, Prevenetive Medicine and Nutrition Clinic, University of Crete, Heraklion, Crete, Greece; 5 Association for Cardiac Research, Rome, Italy

## Abstract

We aimed at performing a calibration and re-calibration process using six standard risk factors from Northern (NE, N = 2360) or Southern European (SE, N = 2789) middle-aged men of the Seven Countries Study, whose parameters and data were fully known, to establish whether re-calibration gave the right answer. Greenwood-Nam-D’Agostino technique as modified by Demler (GNDD) in 2015 produced chi-squared statistics using 10 deciles of observed/expected CHD mortality risk, corresponding to Hosmer-Lemeshaw chi-squared employed for multiple logistic equations whereby binary data are used. Instead of the number of events, the GNDD test uses survival probabilities of observed and predicted events. The exercise applied, in five different ways, the parameters of the NE-predictive model to SE (and vice-versa) and compared the outcome of the simulated re-calibration with the real data. Good re-calibration could be obtained only when risk factor coefficients were substituted, being similar in magnitude and not significantly different between NE-SE. In all other ways, a good re-calibration could not be obtained. This is enough to praise for an overall need of re-evaluation of most investigations that, without GNDD or another proper technique for statistically assessing the potential differences, concluded that re-calibration is a fair method and might therefore be used, with no specific caution.

## Introduction

Re-calibration is the statistical process which allows to adapt a risk function applied to a different population in view of eliminating the over- or under-estimation of risk in the importing population^1,2^. This has become a common practice in cardiovascular risk estimation when some research groups, such as the Euro-Score group and the Framingham research group, have applied their risk functions to other countries or populations. Calibration describes how accurately estimates of prediction of survival (or mortality or incidence) from a model reflect the survival (or mortality or incidence) in the observed data. Therefore, it is an index of accuracy. A well calibrated risk score or prediction rule assigns correct event probabilities at all levels of predicted risk. A miss-calibrated process under- or over-predicts the event probabilities, sometimes globally (calibration at large), sometimes depending on risk levels of specific covariates.

The reason for using re-calibration procedures is that in some countries there are no valuable population studies to produce local risk functions. On the other hand, this acknowledges the fact that applying risk functions of the derivation population may produce gross over- or under-estimation of risk in the validation population. This fact was first demonstrated many years ago^3^, and re-documented more recently^4^, by the Seven Countries Study of Cardiovascular Diseases, where risk functions for coronary heart disease (CHD) incidence or mortality derived from Northern European (NE) or North American populations grossly over-estimated the risk when applied to Southern European (SE) populations, and vice-versa. This problem was basically ignored by those who proposed the so called European coronary risk chart in 1994^5^ using the Framingham Heart Study predictive models, that have repeatedly been shown to over-estimate coronary risk in Europe^6–9^. It also became the reason why, years later, the Euro-Score research group was eventually compelled to produce two different charts for CHD mortality prediction in Europe, corresponding to high risk and low risk populations respectively^10^. On the other hand, a number of contributions were published in the last 15 years^11–23^ that, in the majority, claimed successful usually starting from the assumption that the “imported” coefficients should be valid for the validation population.

The purpose of this analysis was to perform a calibration and re-calibration process using data from two population groups whose parameters and data were all fully known, so that we could establish whether re-calibration gave the right answer. Moreover, we took advantage by the use of a recently proposed technique for assessing statistically calibration outcome that was specifically designed and tested for material consisting of survival data^24^.

## Material and Methods

Population samples used for this analysis derived from the Seven Countries Study of Cardiovascular Diseases. We selected two rural sample in Finland (East and West Finland) and one in the city of Zutphen, the Netherlands that were pooled together to represent Northern Europe (NE). Then, we selected two rural samples in Italy (Crevalcore and Montegiorgio) and two rural samples in Greece (Crete and Corfu) that were pooled together to represent Southern Europe (SE). The entry examination was held between 1959 and 1961 with the enrolment of 2555 middle-aged men in NE and 2927 men in SE, representing respectively 93% and 98% of defined samples. For some purposes of the analysis, data of the US sample and the pool of the three Serbian samples of the Seven Countries Study were also used, as described later. Details on the Seven Countries Study can be found elsewhere^25^.

Six risk factors used for this analysis were: a) age in years, approximated to the nearest birthday; b) body mass index in kg/m^2^, derived from the measurement of height and weight following the technique described in the WHO Manual Cardiovascular Survey Methods^26^ (WHO Manual); c) smoking habits, derived from a questionnaire, classified in three variables as never smokers, ex-smokers and smokers treated as dummy variables (0–1), with never smokers used as reference in the multivariable models; d) systolic blood pressure in mm Hg, measured following the technique of the WHO Manual^26^, in supine position, with mercury sphygmomanometer: two consecutive measurements were averaged and used for analysis; e) heart rate in beats/min, derived from a resting ECG tracing; f) serum cholesterol in mmol/L, measured on casual blood samples following the technique of Anderson and Keys^27^.

All methods were carried out in accordance with relevant guidelines and regulations at the time of the field examinations, performed in the late 50′ to early 60′. In particular, since baseline data were collected before the era of the Helsinki Declaration (June 1964), with consent implied by participation in the examinations, there were no expressed approvals from institutional or licensing committees. However, there were subsequently verbal or written consents given by all informed participants to collect follow-up data.

Mortality data were collected systematically including the availability of deaths certificates plus other information derived from clinical records, interviews with family and hospital physicians, and relatives of the deceased, and any other witness of the fatal event. Coding was made by a single reviewer, following defined rules and criteria and employing the 8th Revision of the WHO ICD^28^. In case of multiple causes of death (present in about half of cases) and of uncertainties about the principal cause of death, a rank order was adopted with violence, cancer, CHD, stroke and other in sequence. CHD was defined by ICD codes 410–413 and cases of sudden death when other specific causes could be reasonably excluded. Cases described as chronic CHD or hypertensive heart disease and cases manifested only as heart failure, arrhythmia or blocks were not coded as CHD for reasons given elsewhere^29^.

Despite the availability of 50-year follow-up data, this analysis was confined to the first 25 years since usually predictive tools of CHD events do not consider too long follow-up periods. Subjects carrying a CHD at baseline were excluded from analysis, as well as those with missing risk factor measurements, thus reducing the denominators to 2360 in NE and 2789 in SE.

Cox proportional hazards models were computed separately for NE and SE using the six risk factors as predictors and 25-year CHD deaths as end-point. Calibration of the models were made using the procedure of Greenwood-Nam-D’Agostino as modified by Demler^24^ (GNDD) that produces chi-squared statistics using 10 deciles of observed and expected (estimated) risk. This approach represents a sophisticated procedure to be used when the material includes survival data and corresponds to the Hosmer-Lemeshaw chi-squared employed for the multiple logistic equation that deals only with binary data. Instead of the number of events, the GNDD test uses survival probabilities of observed and predicted events.

Calibration was performed on the original models (called SELF) separately for NE and SE, by comparing observed and expected risk in decile classes of risk, tested with the GNDD procedure (SELF model). A simulation of re-calibration was carried out for the NE group (validation population) applying the SE (derivation population) risk function components in five different ways, producing five different re-calibrated models: the same was done applying the NE risk function to the SE data. The five re-calibrated models were as follows:

1. model SCM applying the whole SE risk function, including (S) (baseline cumulative survival, S_(0)_), C (coefficients) and M (mean of risk factor levels);
2. model CM substituting only C (coefficients) and M (mean of risk factor levels) of the opposite group;
3. model C, substituting only C (coefficients);
4. model WC, substituting in both areas the original coefficients with another set of coefficients choosing the largest ones -for each risk factor- among those available in the original SE and NE models and in models derived from other cohorts of the Seven Countries Study using the same baseline risk factors, the same end-points and the same length of follow-up; the cohorts were the US Railroad cohort (N = 2406, cases = 373, rate 154 per 1000) and the pool of the three Serbian cohorts of Velika Krsna, Zrenjanin and Belgrade (N = 1540, cases = 133, rate 86 per 1000); the so-called widest coefficients (WC) were not significantly different from those of the original models of both SE and NE groups;
5. ALTS model (altered S_(0)_) computing the chi-squared of the GNDD test after having made artificial (voluntary subjective but very small) changes of the original S_(0)_ of the two population groups; a small increase was considered for NE (from 0.7698 to 0.8098), and a small decrease for SE (from 0.9354 to 0.9254).

The risk probabilities predicted by the models in decile classes of risk were computed and used for the final estimate of the GNDD chi-squared. The above procedures represent an extreme not previously tested and special case (since practically unique) of re-calibration where the parameters of both derivation and validation populations are fully known.

## Results

Table 1 reports mean levels of risk factors, and numbers and rates of CHD fatal events. Risk factor levels of systolic blood pressure, serum cholesterol, and smoking habits were significantly higher in NE than in SE, while BMI was so in SE versus NE, and no significant difference was found for age and heart rate (although the latter close to significance). Death rate from CHD in 25 years was roughly three-fold higher in NE than in SE.

**Table 1:** Mean levels of risk factors and CHD mortality in Northern and Southern Europe.

	NE	SE	P of difference (<)
	Mean and (SD)	Mean and (SD)	
Age, years	49.3 (5.54)	49.14 (5.31)	0.2619
Body mass index, kg/m^2^	23.8 (3.05)	**24.0 (3.67)**	0.0001
Systolic blood pressure, mm Hg	143.7 (20.08)	140.3 (20.97)	0.0001
Heart rate, beats/min	69.1 (13.07)	68.4 (13.10)	0.0632
Serum cholesterol, mmol/L	6.5 (1.32)	5.25 (1.09	0.0001
Never smokers, proportion (*)	15.3 (0.74)	25.0 (0.82)	0.0001
Ex-smokers, proportion (*)	18.0 (0.79)	14.3 (0.66)	0.0001
Smokers, proportion (*)	66.7 (0.97)	60.7 (0.93)	0.0001
CHD fatal CHD in 25 years, N	465	165	—
CHD fatal events, per 1000 in 25 years	197	59	0.0001

NE = Northern Europe; SE = Southern Europe

SD = standard deviation

(*) for proportion = % and (standard error).

Cox models for the two groups (Table 2) showed that BMI had not significant coefficient in NE, but significant in SE, while heart rate and ex-smokers had not significant coefficients in both groups. Age, systolic blood pressure, smokers and serum cholesterol had significant coefficients in both groups. The comparison between the two areas showed a large difference in the baseline cumulative survival that contrasted with the absence of significant differences of risk factors coefficients.

**Table 2:** Cox proportional hazards models predicting 25-year CHD mortality as a function of 6 risk factors in Northern and Southern Europe.

	Delta for Hazard Ratios	NE	SE	P of difference between coefficients (*)
		Hazard Ratios and (95% CI)	Hazard Ratio and (95% CI)	
Age, years	5	1.36 (1.25–1.49)	1.35 (1.15–1.57)	0.8929
Body mass index, kg/m^2^	3	1.01 (0.92–1.11)	1.14 (1.00–1.29)	0.1200
Systolic blood pressure, mmHg	20	1.39 (1.28–1.52)	1.42 (1.22–1.65)	0.8549
Heart rate, beats/min	13	1.02 (0.93–1.12)	1.14 (0.99–1.32)	0.2014
Serum cholesterol, mmol/L	1	1.23 (1.15–1.31)	1.25 (1.10–1.43)	0.8051
Never smokers, proportion	reference	—	—	—
Ex-smokers, proportion	1	1.15 (0.83–1.61)	1.31 (0.75–2.26)	0.7042
Smokers, proportion	1	1.52 (1.16–2.00)	2.02 (1.35–3.02)	0.2570
S_(0)_ = baseline cumulative survival	—	0.7698	0.9354	

NE = Northern Europe; SE = Southern Europe

CI = confidence intervals

Deltas for Hazard Ratios (HR) roughly correspond to 1 standard deviation of each risk factor.

(*) Coefficients not reported.

An intermediate finding of the GNDD procedure is represented by the estimates of risk probabilities for observed and predicted (estimated) events in decile classes of risk. A selection of these data (Table 3) indicated that the NE re-calibrated risk probabilities were similar to the original ones (SELF), only in the model that substituted C alone, and the same was the case for SE. Again, in NE models SCM and CM produced gross under- and over-estimates of risk, respectively, while the reverse was the true for SE. Also the WC and the ALTS models were miss-calibrated.

**Table 3:** Estimated probabilities of CHD mortality risk in deciles of estimated risk from six different models in Northern and Southern Europe. (1 calibration, 5 re-calibration).

Model	Details	Estimated risk probabilities	
		NE	SE
Calibrated SELF	Mean observed	0.2519	0.0801
	Mean expected	0.2555	0.0829
	Decile 1	0.0939	0.0198
	Decile 10	0.5532	0.2452
	Ratio decile10/decile1	5.89	12.39
	p of chi squared of GNDD test	0.7296	0.5942
Re-calibrated SCM	Mean expected	0.1148	0.1908
	Decile 1	0.0311	0.0652
	Decile 10	0.3107	0.4420
	Ratio decile10/decile1	10.0	6.78
	p of chi squared of GNDD test	**<0.0001**	**<0.0001**
Re-calibrated CM	Mean expected	0.3516	0.0547
	Decile 1	0.1161	0.0171
	Decile 10	0.3516	0.1419
	Ratio decile10/decile1	6.40	8.32
	p of chi squared of GNDD test	**<0.0001**	**<0.0001**
Re-calibrated C	Mean expected	0.2624	0.0768
	Decile 1	0.0802	0.0243
	Decile 10	0.6070	0.1957
	Ratio decile10/decile1	7.60	8.06
	p of chi squared of GNDD test	0.2820	0.3776
Re-calibrated WC	Mean expected	0.2763	0.0938
	Decile 1	0.0628	0.0150
	Decile 10	0.7129	0.3295
	Ratio decile10/decile1	11.34	21.94
	p of chi squared of GNDD test	**<0.0001**	**0.0178**
Re-calibrated ALTS	Mean expected	0.2141	0.0950
	Decile 1	0.0765	0.0229
	Decile 10	0.4803	0.2774
	Ratio decile10/decile1	4.32	12.09
	p of chi squared of GNDD test	**0.0265**	**0.0072**

NE = Northern Europe; SE = Southern Europe;

SELF = original model, respectively NE or SE; SCM = substituting S_(0)_, coefficients and means of risk factors; CM = model substituting coefficients and means of risk factors; C = model substituting coefficients only; WC = model substituting coefficients with the widest coefficients that had the corresponding HR (95% CI): age 1.50 (1.28 1.77); BMI 1.21 (1.05 1.41); systolic blood pressure 1.55 (1.32 1.82); heart rate 1.05 (0.81 1.36); cholesterol 1.28 (1.18 1.39); ex-smokers 1.31 (0.75 2.26); smoker 2.18 (1.45 3.28); finally ALTS = substituting S_(0_) with altered S_(0)_: for NE 0.9098 instead of 0.7698; for SE: 0.9254 instead of 0.9354.

GNDD = Greenwood-Nam-D’Agostino-Demler goodness of fit test for re-calibrated survival models: by this test significant difference (outlined here in bold) means that re-calibration is not good enough^24^.

In Table 3 the final outcome of calibration and re-calibration tests are expressed as p of chi-squared according to the procedure of GNDD. In both areas, calibration based on the original (SELF) model was good since associated with high p value of the chi squared. The same happened when only the coefficient of the opposite population were applied, while in all other cases under- or over-estimation or risk were the outcome.

**Figure 1:**
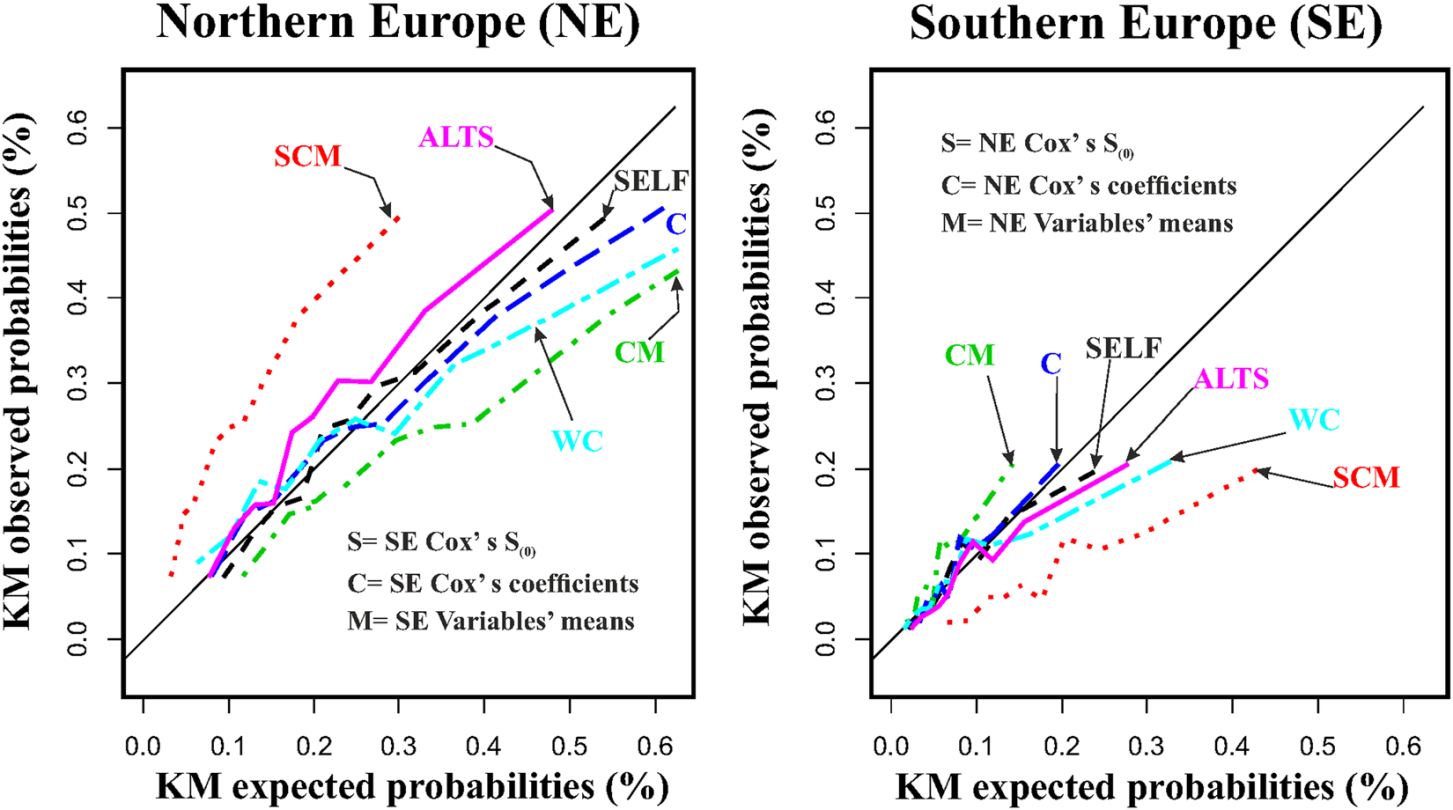
Kaplan-Meier observed versus expected CHD mortality probabilities in Northern (*left:* NE) versus Southern (*right:* SE) Europe in comparison to the identity lines and depending on whether calibration and re-calibration (by five different methods) curves were performed using models: a) with self data (SELF: either NE or SE), corresponding to proper calibration; or re-calibrating by substituting: b1) S_(0)_, coefficients and means of risk factors (SCM); b2) coefficients and means of risk factors (CM); b3) coefficients only (C); b4) deliberately altered S_(0)_ (ALTS); and b5) the widest coefficients (WC) derived from  four population groups of the same study for the six risk factors.

Findings of the above analyses are summarized graphically in Fig. 1 where the SCM model determined an under-estimation of risk in NE and an over-estimation of risk in SE. In the case of model CM an over-estimation was seen in NE and an under-estimation in SE. These effects were mainly due to the fact that the magnitude of coefficients in SE was slightly larger than in NE, despite the absence of significant differences between the pairs of coefficients. On the other hand, a good estimation of risk was seen for the SELF (original) model and model C. Finally, the two models with wide coefficients (WC), and altered S_(0)_ (ALTS) were also miss-calibrated.

## Discussion

### Strengths and limitations

There was no need to produce a re-calibration in these two population groups since for both of them all the data and parameters of the predictive models were fully known, including risk factor levels, identification of events, cumulative survival and multivariable coefficients of the six risk factors. The exercise was directed to apply, in different ways, the parameters of the predictive model of one population to the other (and vice-versa) with the purpose to compare the outcome of the simulated re-calibration with the real data. The main conclusion was that a good re-calibration could be obtained only when we substituted the risk factor coefficients since they were similar in magnitude and not significantly different between the two populations. In all other cases, a good re-calibration could not be obtained.

This exercise has several strength points since the two population groups were studied by the same team within the same cooperative study, examining population samples of the same sex and age range and in the same years, using the same risk factor measurement techniques, the same collection of data procedure, the same follow-up duration, the same diagnostic criteria for end-points and the same coding rules. By contrast, the two population groups had different levels of some major risk factor such as blood pressure, serum cholesterol and smoking habits risk, different rates of CHD mortality, but eventually similar (and not significantly different) multivariable coefficients of risk factors. Moreover, the analysis took advantage from the use of the most recent and sophisticated procedure for estimation of calibration and re-calibration^24^ that takes into proper account the presence of survival data, a technique that was not at hand of previous Investigators during the years 2001–2015 when they claimed in different datasets that re-calibration is possible without specific reserves^11–23^. The only common outcome to all models was an increasing estimated risk from decile 1 to decile 10 which says that the estimate of relative risk could be relatively good, although usually smaller than that provided by the “SELF” (original) models as seen here.

It should be made clear that the SCM model was not the outcome of a real re-calibration but simply a test to explore what happened with a re-calibration procedure. Moreover, it was not granted that by applying the coefficients of the opposite population produced a good re-calibration. This probably happened only because the coefficients of the two populations were very similar. In fact, when we applied the co-called Widest coefficients, based on an arbitrary choice (derived however from data of the same study) the re-calibration was not successful, although the coefficients were not significantly different from the original ones. Similarly, the use of slightly different baseline cumulative survival was an arbitrary selection showing however that by little variations of S_(0)_ outcome might be largely influenced.

There are several limitations in this analysis. In fact, it deals only with male sex and with a restricted age range at entry examination. The analysis was limited to few risk factors and to a limited group of population samples, with findings that cannot be automatically extrapolated to other situations. Moreover, it can be argued that our conclusions cannot be transferred to the present days since population risk factor levels are likely different from those of 50 years ago, also due to the more common drug treatment of high blood pressure and high cholesterol levels. However, this is not demonstrated and if the levels attained by drug treatment have a predictive value, we should not necessarily negate a possible general rule of the relationship between risk factors and CHD events. In fact, it is possible that the slope of the relationship should not depend from risk factors distribution.

On the basis of this experience, we feel that the re-calibration process should be carried with much caution owing to a number of uncertainties that include the relationship of population sample risk factors with the events collected from other sources and the magnitude of the risk factor coefficients coming from other population experiences. This is enough to praise for an overall need of re-evaluation of most investigations^11–23^ that, without a proper technique for statistically assessing the potential differences^24^, concluded that re-calibration is a fair method and might therefore be used, with no specific caution. In general it appears that in order to have a good re-calibration one needs: A) a very good (and/or very lucky) estimate of baseline cumulative survival S_(0)_ that is probably hard to obtain since usually derived from external data; indeed, the estimate of the baseline cumulative survival, usually derived from sparse and diverse sources, cannot be fully trusted to reflect the reality of the study population; B) a set of “imported” coefficients not significantly different from the theoretical (and unknown) ones, but also very similar in magnitude.

### Contributions from the literature

Most of the re-calibration procedures found in the literature^11–23^ started from the assumption that the “imported” coefficients were valid and similar or not significantly different from those (unknown) of the “importing” (validation) populations. These studies were carried in 13 different countries (9 European and two Asian countries, plus Australia and the USA). Several contributions have put together three components derived from largely different sources: risk factor distribution from population samples, mortality data from regional or national origin (not related to a real follow-up) and coefficients from the Euro-Score project or the Framingham Heart Study^14,15,17–21^. Only three studies^11,12,22^ have reported the risk function of the “validation population”, while others did not do so although it was clear that it could have been computed and presented^13,16,19,20,23^. This was particularly unfortunate since a good occasion was lost to investigate the problem of the possible homogeneity of risk factor coefficients. Some contributions have openly claimed that the possible heterogeneity of multivariable coefficients was not a problem, quoting references that did not fully support this concept^13,18^. However, when both the high and low risk functions of the Euro-Score project were tested, the high-risk model did not produce good calibration^13,16,20,21^. This may depend upon a casual combination of high levels of risk factor coefficients and high levels of estimated survival that may tend to balance one each other. In fact, in a test made on our NE population group we found that applying the widest coefficients (that by themselves induce a poor calibration) together with higher levels of survival (that by themselves induce a poor calibration) ended up in an acceptable calibration.

In at least three contributions the Hosmer-Lemeshaw chi-squared test was applied to data derived from models that included survival data^19,21,23^, that is in an improper way.

### Conclusions

On the basis of this experience, good re-calibration can be achieved only in a few cases and when lucky circumstances do coincide. Therefore, much caution must be used in reaching valid conclusions.

The re-calibration procedure will probably lose its role when more and more countries will be able to produce their own risk functions. However, this process may stimulate the need to study deeper the problem of the heterogeneity or non-heterogeneity of multivariable coefficients of risk factors. The absence of systematic differences across multivariable coefficients of CHD risk function has been repeatedly shown comparing the cohorts and/or areas of the Seven Countries Study^3,29–34^ but this seems to be an isolated effort that calls for a systematic approach from many different sources. There is a theoretical, and not only practical issue, since demonstrating the overall similarity in the magnitude of multivariable coefficients could represent the possible identification of a general biological rule linking risk factor levels and events, when everything else being equal.

This analysis was mainly a methodological exercise, but we acknowledge the fact that a number of “new risk factors” may improve the prediction of coronary events. Among others, one may wish to explore the effects of statins on cardiovascular outcomes by smoking status^35^, vitamin-D deficiency and non-lipid biomarkers of cardiovascular risk^36^, evidence-based assessment of lipoprotein(a) as a risk biomarker for cardiovascular diseases^37^, association of serum lipids and CHD in just observational studies^38^ and whether low high-density lipoprotein-cholesterol should be treated^39^. Unfortunately, all these terms either were not available in our old study or the questions were far from our main objective.

### Declarations

#### Ethics approval and consent to participate

Entry examination was held well before the Helsinki declaration with consent implied by participation in the examinations, while subsequently verbal or written consent was obtained to collect follow-up data.

#### Availability of data and materials

The data are not available to the public or shared as they are still confidentially kept by the Principal Investigators of the Seven Countries Study.
